# Epidemiology, injury mechanisms, and prevention strategies in Ultimate Frisbee

**DOI:** 10.3389/fspor.2026.1714194

**Published:** 2026-05-04

**Authors:** Yongxia Chen, Jie Gao, Pengquan Zhang, Jingyi Yan, Xin Wang

**Affiliations:** 1 College of Exercise Health, Shenyang Sport University, Shenyang, China; 2 College of Winter Sports, Shenyang Sport University, Shenyang, China; 3 Boratory Management Center, Shenyang Sport University, Shenyang, China

**Keywords:** injury mechanisms, injury prevention, sports epidemiology, sports medicine, Ultimate Frisbee

## Abstract

Ultimate Frisbee, a high intensity noncontact sport, poses significant injury risks due to the dynamic demands of sprinting, cutting, and jump landings. These injuries negatively impact athletic performance and carry potential long term consequences. Despite rapid global growth, current epidemiological data exhibit significant methodological variance, and the precise biomechanical mechanisms remain insufficiently classified. Consequently, this study systematically reviews the epidemiology and mechanisms of sports injuries in Ultimate Frisbee while narratively synthesizing sport specific prevention and rehabilitation strategies extrapolated from analogous high demand sports. Researchers systematically screened relevant literature from databases including PubMed, Web of Science, SPORTDiscus, the Cochrane Library, CNKI, Wanfang, VIP, and Google Scholar published between January 1990 and March 2025. The synthesized evidence indicates a profound predominance of lower extremity trauma including lateral ankle sprains, knee ligament tears, and hamstring strains. Conversely, upper extremity, trunk, and neurological traumas including concussions frequently result from aerial contests and high impact layouts. Because direct interventional evidence in Ultimate Frisbee remains scarce, mitigating these risks requires adopting targeted neuromuscular protocols, optimizing cleat surface mechanical interactions, and leveraging wearable technologies for workload prediction. Furthermore, post injury management necessitates the implementation of individualized rehabilitation protocols and objective functional metrics to effectively dictate safe return to play. This review establishes a comprehensive conceptual framework bridging epidemiological data with biomechanical mechanisms, ultimately guiding clinical practitioners and coaches in developing targeted risk management and performance optimization protocols.

## 1 Introduction

With the increasing intensity of competitive sports, sport-related injuries have become a major determinant of athletes' career longevity. As modern participation diversifies, Ultimate Frisbee has attracted growing attention: since being included in the World Games in 2001, it has spread rapidly worldwide, and was highlighted by The New York Times in 2009 as one of the world's fastest-growing sports (1). However, research on injury prevention and risk management in Ultimate Frisbee has lagged behind its rapid development. In particular, the discontinuity of injury surveillance and the lack of validated, sport-specific prevention protocols warrant urgent attention. Clarifying the sport's injury profile and mechanisms to guide evidence-based interventions is therefore critical for improving athlete welfare and competition quality. Addressing these needs, the present review synthesizes existing literature to delineate major injury patterns, injury mechanisms, risk factors and potential interventions, with the aim of providing a theoretical basis for developing targeted injury prevention strategies and practical guidelines.

Ultimate Frisbee (often simply referred to as Ultimate) is a high-intensity, officially non-contact team sport centered on passing and catching a flying disc, incorporating tactical elements of basketball, soccer, and American football (2–4). Current research indicates that Ultimate Frisbee features external load characteristics such as high-speed running, rapid stop-start actions, and quick changes of direction, compounded by aerial contests and incidental contact (5–8). These demands impose substantial shear, rotational, and eccentric braking stresses on the lower extremities, during decelerating, cutting, and landing jumps (9, 10). Such forces place significant strain on musculoskeletal structures, contributing to persistently high lower-limb injury rates—most commonly ankle sprains, knee ligament injuries, and quadriceps/hamstring strains (11, 12). Physiological monitoring characterizes Ultimate Frisbee as a “high-intensity, high-frequency, high-fatigue” sport, where players sustain mean heart rates of 82% HRmax (peaking at 99%) and cover over 0.8 km via high-speed sprinting (11). Research on elite female athletes indicates a mean metabolic power of 12.2 ± 1.7 W/kg and an intermittent index of 1.24, suggesting that frequent accelerations impose loads that traditional speed metrics may underestimate (12). Additionally, data from national championships reveal that while athletes spend approximately 63% of match time at high intensities (>70% HRmax), significant physiological declines occur in later tournament stages, highlighting the critical challenge of cumulative fatigue (13). In this context, the sport demands frequent high-power bursts and the capacity for rapid acceleration and deceleration; if preparation or recovery is inadequate (14), cumulative eccentric braking loads and compromised neuromuscular control may elevate fatigue and lower-limb injury risk (15).

Although Ultimate Frisbee is designated as non-contact (16), the explosive movements involved (e.g., sprinting, cutting, aerial catches) confer a substantial injury risk. Epidemiological evidence from prospective studies indicates substantial injury risks, with incidence rates ranging from 9.6 to 33.4 per 1,000 athlete-exposures (AE) in collegiate and professional settings, and estimates reaching as high as 84.9 when including all injury time-outs (15, 17–19). Injuries predominantly involve the lower limbs, specifically the knee, thigh, and ankle, with muscle injuries and joint sprains representing the leading clinical diagnoses (17, 19). By injury mechanism, approximately 29% to 36% of injuries are due to direct contact with other players, while the remaining injuries are attributed to non-contact events. This highlights the dual risks associated with high-speed locomotion and situational contact (15, 17, 19). As participation and performance levels increase, the need for injury prevention becomes increasingly urgent. However, systematic research on the sport's injury characteristics, mechanisms, and prevention strategies remains scarce. Much of the existing literature relies on case reports or anecdotal summaries, lacking robust, detailed empirical data. Furthermore, updated research is limited and evidence-based guidelines specifically tailored to the evolving competitive demands and diverse athlete populations are lacking.

**Figure 1:**
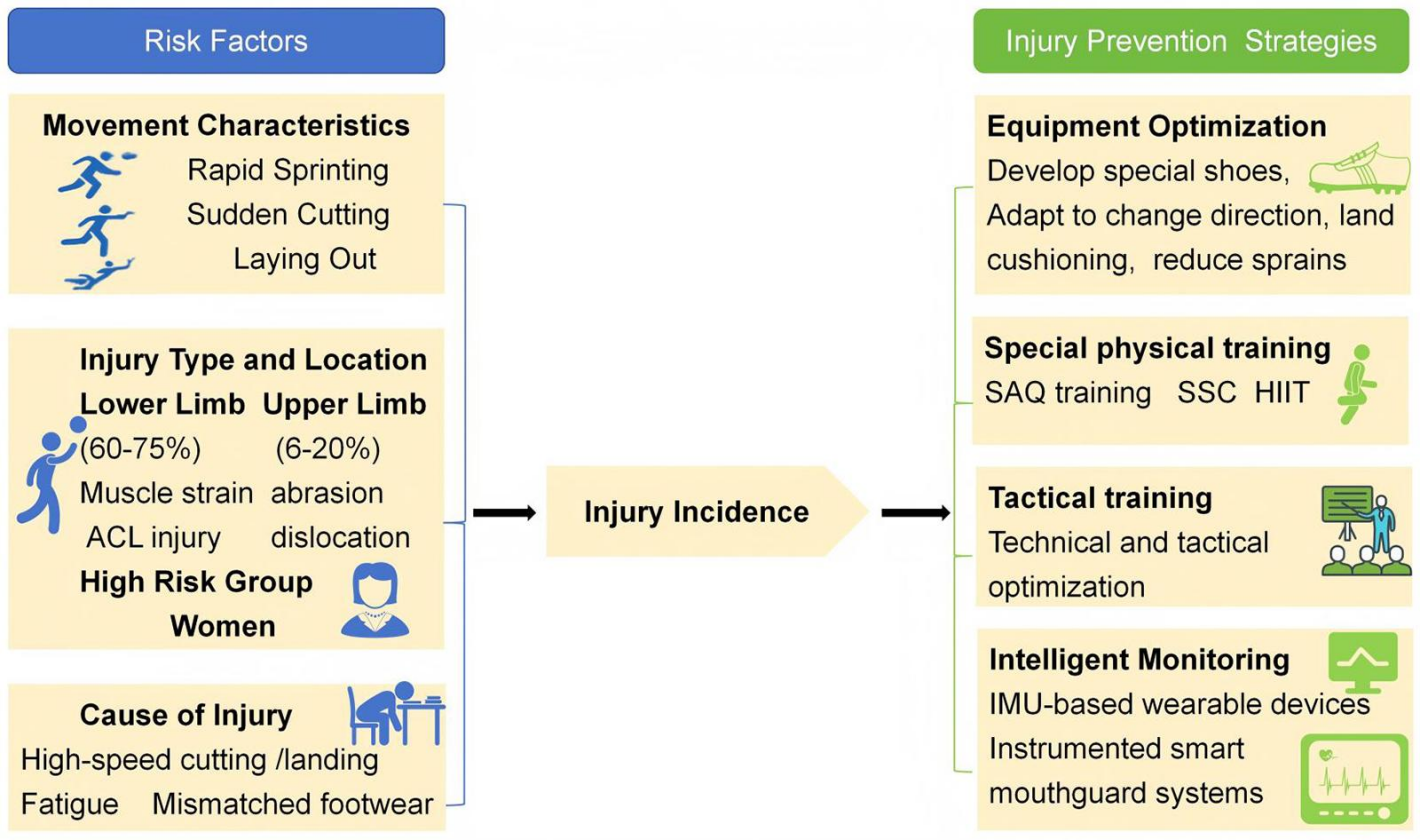
Mechanisms of injury and comprehensive prevention pathways in Ultimate Frisbee. A conceptual framework of sports injuries in Ultimate Frisbee encompassing epidemiology, mechanisms, prevention strategies, and clinical rehabilitation. Items included in this diagram highlight the most prevalent pathologies and the most clinically significant interventions specific to the unique biomechanical demands of the sport rather than serving as an exhaustive medical list.

This review systematically synthesizes 35 years of injury research in Ultimate Frisbee utilizing a comprehensive framework encompassing epidemiology, injury mechanisms, prevention strategies, and clinical rehabilitation, as illustrated in Figure 1, to systematically identify potential risk factors and management pathways in the sport. We examine injury incidence, common pathologies, and anatomical distributions, delineate injury severity, mechanisms, and associated risk factors, and evaluate the efficacy and limitations of current prevention strategies. Based on this synthesis, we propose future research directions aimed at providing a theoretical basis and practical guidance for evidence-based conditioning and targeted risk management.

## 2 Materials and methods

### 2.1 Study design

This study was designed as a narrative review with a systematic search strategy. To ensure methodological rigor and transparent reporting, the review process was conducted in accordance with the PRISMA (Preferred Reporting Items for Systematic Reviews and Meta-Analyses) guidelines where applicable (20–22). The review protocol was not prospectively registered in PROSPERO; however, the search strategy, inclusion criteria, and data synthesis methods were established *a priori* to minimize selection bias.

### 2.2 Search strategy

Systematic searches were conducted in PubMed, Web of Science, SPORTDiscus, the Cochrane Library, CNKI, Wanfang, VIP, and Google Scholar. The search period covered literature published from January 1, 1990, to March 31, 2025. The search strategy employed a combination of medical subject headings (MeSH) and free-text terms. Keywords in English included “Ultimate Frisbee,” “Flying Disc,” “injury,” “epidemiology,” “prevention,” “sports injury,” and “risk factors.” Chinese-language terms, including “极限飞盘” (Ultimate Frisbee), “飞盘运动” (Flying Disc), “损伤” (injury), “流行病学” (epidemiology), “预防” (prevention), and “风险因素” (risk factors), were also utilized. In addition to database searches, backward citation tracking was performed on relevant reviews and eligible articles to maximize the retrieval of pertinent studies.

### 2.3 Inclusion and exclusion criteria

#### 2.3.1 Inclusion criteria

Studies were included if they met the following criteria: (1) original research involving Ultimate Frisbee athletes (recreational, collegiate, or professional); (2) studies reporting quantitative data on injury incidence, types, mechanisms, or risk factors; (3) observational (cohort, cross-sectional) or interventional study designs; and (4) articles published in peer-reviewed journals.

#### 2.3.2 Exclusion criteria

Studies were excluded if they: (1) studies strictly limiting injury definitions to “time-loss” only without reporting medical attention cases, as these may significantly underestimate the injury burden in non-contact sports; (2) non-original research (e.g., reviews, editorials, letters); (3) were conference abstracts, theses, or gray literature lacking sufficient methodological detail; or (4) studies where Ultimate Frisbee data could not be disaggregated from other sports.

#### 2.3.3 Study selection process

**Figure 2:**
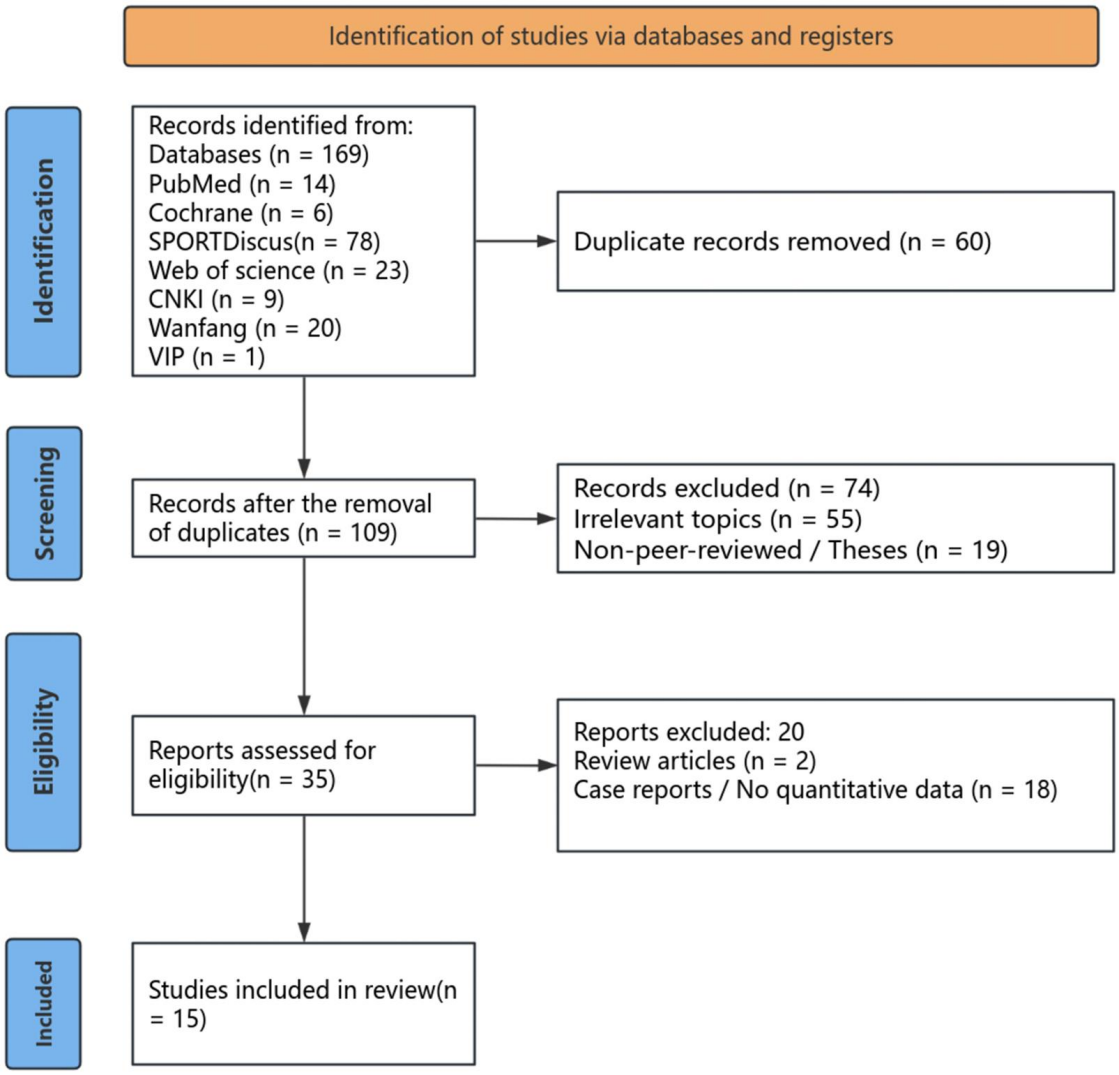
PRISMA 2020 flow diagram describing the literature search and study selection process. The database search included PubMed, Web of Science, SPORTDiscus, Cochrane Library, Google Scholar, CNKI, Wanfang, and VIP. n, number of records; PRISMA, Preferred Reporting Items for Systematic Reviews and Meta-Analyses.

Two researchers independently screened titles and abstracts. Full texts of potentially eligible studies were retrieved and reviewed. Any discrepancies regarding study inclusion were resolved through discussion or adjudication by a third researcher. The detailed screening process and reasons for exclusion are presented in the PRISMA flow diagram (Figure 2).

### 2.4 Quality assessment

The methodological quality of the 15 included studies, spanning a publication period from 1990 to 2025, was rigorously evaluated using the JBI Critical Appraisal Checklist for Studies Reporting Prevalence Data. The assessment focused on four key domains: (1) appropriateness of the sample frame and sampling method; (2) adequacy of sample size; (3) validity of injury identification methods, distinguishing between medical diagnosis and self-reported data; and (4) standardization of statistical analysis.

## 3 Results

### 3.1 Study characteristics and methodological quality

The included literature reflects the global expansion of the sport, evolving from early research in the United Kingdom (23) and North America (19, 24) to recent epidemiological data from Asia (25–27). The included studies also represent a wide spectrum of competitive levels, ranging from elite professional tiers (15) and national championships (19, 28) to the predominant collegiate and university club sectors (17, 18, 29). Despite this geographical and demographic diversity, the synthesis of data is challenged by significant methodological heterogeneity. A primary source of variation lies in the study design and its implications for recall bias. Only a minority of the included studies utilized rigorous prospective cohort designs (15, 17–19). By tracking athlete-exposures (AEs) in real-time, these studies provided high-quality incidence data with minimal bias (15, 19). In contrast, the majority of the literature consists of retrospective cross-sectional designs (10, 14, 24, 25, 29). While instrumental in describing broader injury distributions, these studies are susceptible to significant recall bias, potentially underreporting minor injuries or overestimating the prevalence of severe, memorable incidents.

### 3.2 Inconsistency in injury definitions

The interpretation of prevalence rates is further complicated by the lack of a standardized injury definition across the included studies. The assessment identified three distinct operational approaches: (1) Time-Loss Definition: Studies employing this criterion strictly defined injury as a condition requiring withdrawal from play or training (17, 19). This rigorous approach yielded more conservative injury rates (approximately 12.6–13.0 injuries per 1,000 AEs) by filtering out minor complaints. (2) Medical Attention Definition: In contrast, Hess et al. (15) adopted a broader definition capturing any complaint requiring medical evaluation, resulting in a significantly higher reported rate (33.4 per 1,000 AEs), reflecting the full clinical burden of the sport rather than just functional disability requiring withdrawal from play. (3) Self-Reported/Subjective Definitions: This lack of clinical standardization is particularly evident in studies relying on participant self-classification, a method that is prone to conflating pathological injuries with non-pathological fatigue or minor delayed onset muscle soreness (24).

In summary, the methodological limitations identified—ranging from the subjective nature of self-reports in specific diagnostic patterns, such as concussion prevalence (30), to the variability in radiological review protocols (8)—were carefully considered during the data synthesis and are further addressed in the discussion.

### 3.3 Injury incidence and prevalence

The epidemiological characteristics, including specific injury definitions and reported incidence or prevalence rates extracted from the 15 included studies, are summarized in Table 1. A critical analysis of these data reveals a distinct methodological divergence between true exposure-based incidence rates derived from prospective monitoring and retrospective prevalence proportions.

**Table 1:** Summary of epidemiological study characteristics, injury definitions, and reported incidence or prevalence rates in Ultimate Frisbee.

Author (Year)	Study design and level	Sample size (*N*)	Injury definition^a^	Incidence rate^b^ (n/1,000 AEs)	Injury prevalence/key proportions^c^ (%)	Quality
Marfleet et al. (23) (1991)	Retrospective (Tournament, UK)	∼1,000 players (485 injuries)	Self-reported (during tournament)	NR	Lower limb: 66.6%; thigh muscle strain: 14.4%; ankle sprain: 10.9%; skin abrasions/friction burns: 14.0%	Low
Reynolds et al. (24) (2006)	Retrospective (Club, USA)	135 respondents	Self-reported (any injury)	NR	Muscle strain: 76%	Low/moderate
Yen et al. (19) (2010)	Prospective (Collegiate- USA)	32 teams (705 players)	Time-loss (injury timeout)	ITO: 110 Return prevented: 1.66	Lower extremity: approximately 52% (men 53%, women 51%)	High
Akinbola et al. (29) (2015)	Retrospective (Collegiate, USA)	143 injured cases	Self-reported (any physical complaint)	NR	Knee: 35%	Moderate
					Ankle: 23.1%	
					Hamstring: 7.7%	
Swedler et al. (17) (2015)	Prospective (Collegiate, USA)	106 teams (73 colleges)	Time-loss (missed game/practice)	12.6	Lower extremity: 67%	High
Kołodziej et al. (14) (2018)	Retrospective (Tournament, Poland)	110 players	Self-reported (last 12 months)	NR	100% (all participants reported injuries)	Low/moderate
Lazar et al. (30) (2018)	Retrospective (Competitive, USA)	787 players	Self-reported (concussion focus)	NR	Concussion prevalence: 26.1% (26.6% men, 24.8% women)	Moderate
Hess et al. (15) (2020)	Prospective (Professional, AUDL)	16 teams (approximately 390 players)	Medical attention (evaluated by athletic trainer)	33.4	Thigh strain: 12.7%	High
					Ankle sprain: 11.4%	
Brezinski et al. (18) (2020)	Prospective (Collegiate Club, USA)	196 (total across 6 sports)	Medical attention (by athletic trainer)	Men's UF: 9.6 Women's UF: 11.1	68 injuries	High
Pang et al. (25) (2021)	Retrospective survey (Club/National, HK)	59	Self-reported (calculated via estimated hours)	5.7 (/1,000 h)	Injury prevalence: 62.7%	Moderate
					Lower limb: 61.1%	
Khoo et al. (28) (2021)	Retrospective survey (Elite Club, USA)	56^+^ (injured players)	Self-reported (past season)	NR	Lower extremity: 88% (of injured players)	Moderate
Baharuddin et al. (26) (2022)	Cross-sectional (University, Malaysia)	138	Self-reported	NR	Lower limb: 49.3%	Low/Moderate
					Muscle strains: 28.5%	
Cheng et al. (27) (2022)	Cross-sectional (Club, Malaysia)	211	Self-reported (focus on prevention strategies)	NR	Lifetime injury prevalence: 98.1% (207/211 athletes)	Moderate
Muramoto et al. (10) (2024)	Retrospective survey (College, Japan)	116	Self-reported (past year)	NR	Injury prevalence: 49.1%	Moderate
					Lower limbs: 42/57 injured (73.7%)	
Coulter et al. (8) (2025)	Retrospective radiological review (Clinical, USA)	187 injury encounters	Radiological diagnosis (x-ray, CT, MRI)	NR	Knee: 23.5%	Moderate
					Shoulder: 14.8%	
					Surgery required: 14.4%	

NR, not reported in the original study (typically due to the lack of exposure tracking in retrospective survey designs).

^a^ Injury definition: criteria used by the original authors to define an injury. “Time-Loss” typically requires the athlete to miss a subsequent practice or game; “Medical Attention” includes any complaint evaluated by medical staff regardless of time lost; “Self-Reported” relies on the athlete's subjective recall of pain or dysfunction without formal clinical diagnosis.

^b^ Incidence rate: expressed as the number of injuries per 1,000 Athlete-Exposures (AEs) unless otherwise specified.

^c^ Prevalence/key proportions: represents either the percentage of players who sustained at least one injury (prevalence) or the percentage of total injuries attributed to a specific diagnosis/anatomical location (proportion).

#### 3.3.1 Incidence rates in prospective studies

In sports epidemiology, the gold standard for assessing injury risk is the calculation of incidence rates using “Athlete-Exposures” (AEs). High-quality prospective studies utilizing this metric report incidence rates ranging from 9.6 to 33.34 injuries per 1,000 AEs in Ultimate Frisbee. During the 2017 season, Hess et al. prospectively monitored 16 all-male professional teams in the American Ultimate Disc League (AUDL) and demonstrated a remarkably high overall incidence rate of 33.34 injuries per 1,000 AE (15). To contextualize this magnitude, this rate is comparable to the match injury incidence in professional soccer, which is typically reported around 27.5 injuries per 1,000 h (31, 32). Furthermore, sports like basketball, which share similar biomechanical loads with Ultimate Frisbee, also present significant injury burdens regarding ankle sprains and anterior cruciate ligament (ACL) tears (33–35). However, the overall incidence in Ultimate remains lower than the extreme rates exceeding 80 injuries per 1,000 h seen in heavy collision sports such as Rugby Union (35). These comparisons powerfully underscore that Ultimate Frisbee, despite being a non-contact sport, poses significant injury risks due to the dynamic demands of sprinting, cutting, and jump-landing, which are established risk factors across major field sports (5, 8, 36).

At the collegiate and club levels, researchers utilizing stricter “time-loss” definitions report more conservative figures. Swedler et al. (17) tracked 106 collegiate teams over a season and reported a rate of 12.6 injuries per 1,000 AEs. In a comprehensive study comparing multiple club sports, Brezinski et al. (18) reported rates among U.S. collegiate club players of 9.6/1,000 AE in men and 11.1/1,000 AE in women, these collegiate Ultimate injury rates were generally lower than the rates of collegiate soccer (14.3–19.5 per 1,000 AEs) and American football (25.1 per 1,000 AEs) within the exact same collegiate setting.

#### 3.3.2 Injury prevalence in retrospective surveys

In retrospective survey designs where precise exposure tracking is absent, the reported injury prevalence is notably high. Lifetime or past-year injury prevalence rates reached 98.1% and 100% in specific cohorts (14, 27).

In studies using injury proportions, Reynolds et al. (24) conducted an anonymous retrospective survey among 135 adult players at a regional tournament. They found that 88% had missed training or competition due to injury, 71% had sought medical care, and 49% reported recurrent injuries, indicating a significant overall injury burden. Similarly, Akinbola et al. (29) retrospectively analyzed injury clinic records from collegiate club and performing arts programs (2000–2012), identifying 461 cases of sports-related injuries, of which Ultimate Frisbee accounted for 31% (143/461), second only to rugby (33.8%, 156/461). Furthermore, Khoo et al. (28) surveyed elite U.S. club athletes before and after the 2019 season, reporting that 98% had a history of Frisbee-related injuries prior to the season, while 63% sustained new injuries during the season, most of which involved the lower extremities. While these figures effectively highlight the widespread nature of injuries, the reliance on subjective memory introduces significant recall bias, emphasizing the necessity of utilizing prospective incidence rates for an accurate risk assessment.

### 3.4 Injury sites and sex differences

**Figure 3:**
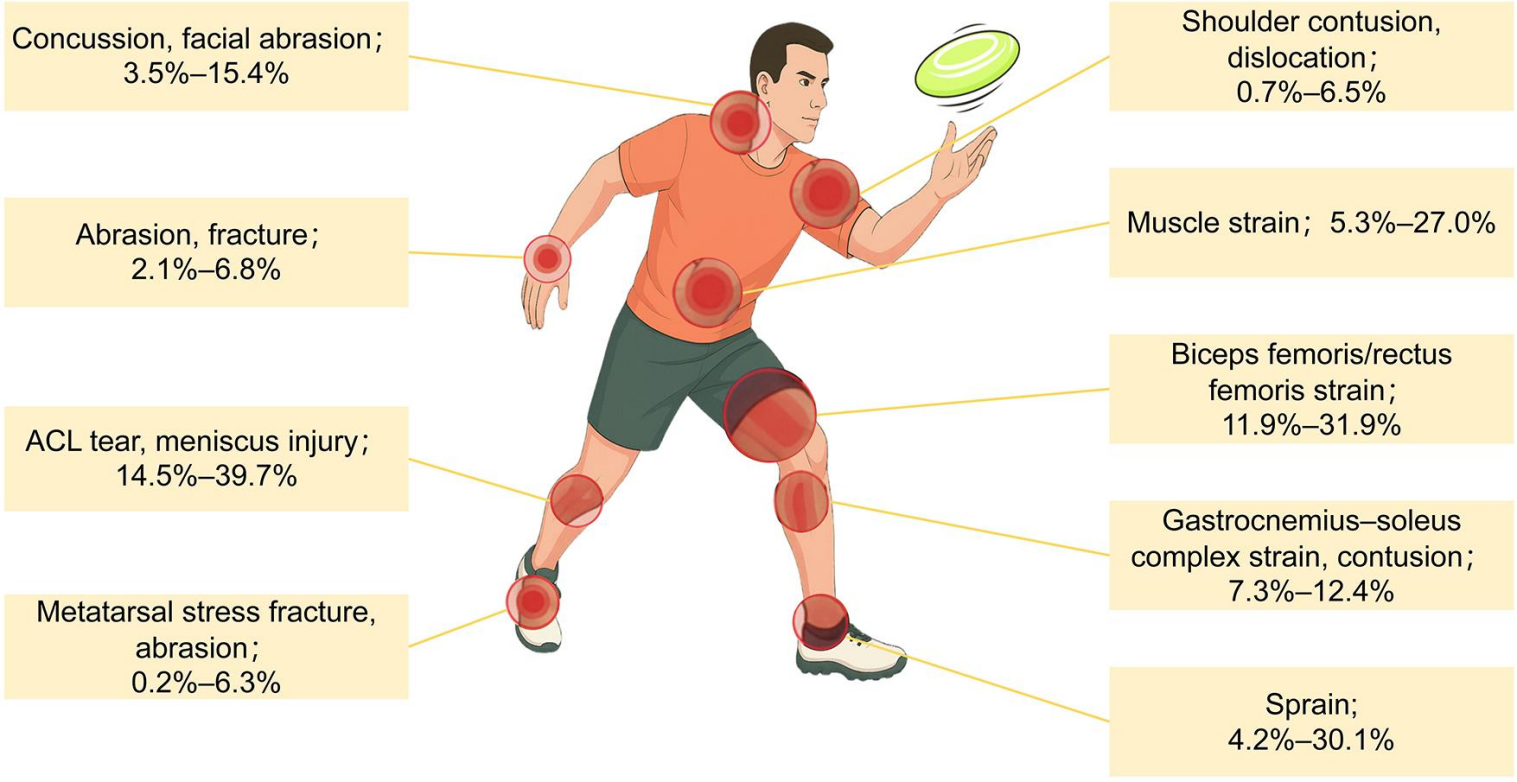
Injury distribution and types in Ultimate Frisbee. Proportional anatomical distribution of sports injuries in Ultimate Frisbee. Data presented represent generalized trends synthesized from the included epidemiological studies, emphasizing the significant predominance of lower-limb trauma across varying methodologies and sample populations.

Based on the synthesized epidemiological data, sports injuries in Ultimate Frisbee exhibit a highly specific anatomical distribution. The overarching trend indicates a profound susceptibility of the lower extremities to non-contact trauma, whereas the upper limbs, trunk, and head are predominantly subjected to contact-related mechanisms during aerial engagements. The generalized proportional distribution of these anatomical injury sites is visually represented in Figure 3. Subsequent sections delineate the specific pathologies and sex-based variances within these distinct anatomical regions.

#### 3.4.1 Lower-limb injuries

Consistent across all geographical regions and competition levels, lower-limb injuries are most prevalent. The unique kinematic profile of Ultimate Frisbee is characterized by abrupt pivoting, high-velocity cutting, and repetitive jump-landing sequences, all of which impose substantial biomechanical loads on the knee and ankle articulations (5, 8).

Several studies corroborate this specific regional dominance. Hess et al. (15) analyzed 299 injuries reported during the 2017 AUDL season and found that 72% involved the lower limbs, with ankle (19%), thigh (17%), and knee (14%) being the most frequent sites, underscoring the high-load and high-exposure nature of the sport for the lower body. Baharuddin's cross-sectional survey of Malaysian university athletes similarly found that lower-limb injuries accounted for 49.3% (26). Furthermore, Muramoto et al. reported that 74% of injuries among Japanese collegiate players involved the lower extremities, including the hip, thigh, knee, calf, ankle, and foot, with the ankle and knee being the most prevalent (10). However, a critical evaluation reveals significant variance in specific diagnoses depending on the data collection methodology. Subjective self-reported surveys often capture an exceptionally high proportion of muscle strains, reaching up to 76% (24). Conversely, standardized radiological data present a more severe clinical reality. A decade-long radiological review found that 14.4% of Ultimate-related injuries required surgical intervention, predominantly involving knee ligamentous trauma at 23.5%, including ACL and meniscal tears (8).

#### 3.4.2 Upper-limb and trunk injuries

Upper-limb and trunk injuries occur less frequently and account for approximately 12.2%–17.5% of total injuries, but they often result from aerial contests and layout maneuvers. The unique mechanics of the layout frequently expose the shoulder girdle to severe ground-impact trauma. Supporting this mechanism, Marfleet et al. (23) reported that 18% of injuries involved the upper limbs, with fractures of the fifth metacarpal being common, often due to improper ulnar-sided landings during layouts.

Regarding trunk involvement, Muramoto et al. (10) noted that back injuries accounted for only 1.8% of all cases among Japanese collegiate athletes, typically associated with falls or collisions. In a Malaysian university cohort, Baharuddin et al. reported that trunk injuries accounted for 7% (26). However, Reynolds et al. (24) found that 21% of adult players surveyed reported rib injuries, suggesting that thoracic involvement should not be overlooked in self-reported data.

Additionally, aerial contests for the disc carry a distinct risk for head injuries. Swedler et al. (17) reported 89 head injuries and 47 concussions based on online surveillance during the 2012 U.S. college ultimate season, corresponding to incidence rates of 0.85 and 0.45 per 1,000 AEs, respectively. Reynolds and Halsmer conducted a retrospective survey of 135 adult ultimate players and found that 30% of respondents had sustained a head injury while playing, a frequency that was lower than that of lower extremity and shoulder injuries (24). Injury surveillance at multiple international tournaments by Marfleet revealed that facial injuries were predominantly contusions and lacerations, typically caused by being struck by the disc, whereas head injuries most often resulted from collisions between players (23). Furthermore, Baharuddin et al. reported that head injuries accounted for approximately 12% of all injuries in their sample of Malaysian university ultimate players (26).

#### 3.4.3 Sex-specific injury patterns

Regarding sex differences in injury risk and anatomical distribution, the synthesized evidence remains complex. Female collegiate players experienced an ACL injury rate 1.5 times higher than their male counterparts (17). This trend is strongly supported by recent studies noting a statistically higher likelihood of non-contact lower-extremity injuries in women (10, 29).

Conversely, Khoo et al. (28) found significantly higher rates of ankle and calf injuries in men among elite club athletes. As noted in current literature, such discrepancies may reflect underlying differences in competitive levels, specific tournament structures, or variations in playing styles. For example, the higher frequency of aggressive aerial contests and layout behaviors in men's divisions often leads to greater impact-related upper body trauma (14, 17, 26). This highlights a critical need for future prospective research to utilize standardized tracking to isolate true sex-related biomechanical risk factors.

## 4 Injury types and mechanisms

Following the International Olympic Committee (IOC) classification standards for sports injuries (37), this review categorizes injuries in Ultimate Frisbee based on affected tissues and pathological types, along with detailed descriptions of representative mechanisms. The sport combines sprinting, jumping, abrupt deceleration, cutting, and aerial catching, which expose multiple body regions to complex mechanical stresses. Evidence indicates that injuries are predominantly concentrated in the lower limbs, with common types including muscle strains, ligament sprains, and joint dislocations, as well as more severe structural injuries such as concussions and fractures (23, 24). These injuries are not only caused by external impacts but are also closely linked to energy transfer, tissue tolerance thresholds, and neuromuscular control during play.

### 4.1 Soft-tissue injuries

Although Ultimate Frisbee is classified as a non-contact sport, the intense demands of cutting maneuvers impose substantial loads on the lower-limb joints, frequently leading to non-contact injuries of the knee ligaments and cartilage. Coulter et al. (8) reported that ACL tears accounted for 20.5% of knee injuries in Ultimate Frisbee. Muramoto et al. (10) further observed that lower-limb injuries were predominantly non-contact, frequently occurring during sprinting, cutting, jumping, and turning. Previous studies have shown that cutting maneuvers in Ultimate Frisbee are characterized by near-right-angle turns (mean cut angle approximately 94°), with entry and exit velocities of approximately 3.4 m/s and 3.7 m/s, respectively, and a mean knee flexion angle of only 34° during stance (9), it has been highlighted that during high-speed, sharp directional changes, insufficient knee flexion can result in increased shear and torsional forces acting on the lower limb joints, thereby elevating the risk of injury to structures such as ACL (9, 38, 39). Previous studies have identified that cutting maneuvers are associated with tri-planar coupled loading during the support phase, which is especially common in sudden direction changes. These include knee valgus coupled with internal rotation of the femur/tibia, particularly during knee flexion (40). This high-risk pattern is not the result of a single factor, but rather emerges from a complex mechanical environment shaped by multiple factors, including the alignment of landing, posture control of the upper and lower limbs, and the characteristics of ground reaction forces during the support phase (41, 42). When landing laterally, the line of action of the ground reaction forces shifts laterally relative to the knee joint center, creating a larger knee abduction moment (43, 44). At the same time, internal rotation of the femur/tibia is commonly observed, leading to combined stresses in the sagittal and transverse planes, which significantly increases strain on the ACL and medial collateral ligament, thus heightening the injury risk (43). Furthermore, Della et al. (45) found that insufficient knee flexion combined with knee valgus, hip internal rotation and adduction, and trunk lean toward the injured side in football players led to a decrease in structural stability, subsequently resulting in ACL rupture. Systematic video analyses from professional rugby union further corroborate this, confirming that non-contact ACL injuries frequently occur during cutting and sidestepping maneuvers when the knee is exposed to combined internal rotation and anterior shear forces (46). Furthermore, similar to jump-landing mechanics observed in netball and soccer, ankle sprains in Ultimate represent another major soft-tissue concern. During jump-landings, the foot is frequently positioned in a highly vulnerable state of combined plantarflexion and inversion upon ground contact. This excessive inversion trauma generates immense biomechanical stress on the lateral ligament complex, primarily leading to structural tears in the anterior talofibular ligament (47–49).

Muscle and tendon injuries are the most common soft tissue traumas in Ultimate Frisbee, particularly involving the hamstrings, rectus femoris, and gastrocnemius (15, 24, 25). These injuries typically occur during sprinting, jump-landing, and abrupt deceleration or cutting. The biomechanical etiology of hamstring strains can be elucidated through running mechanics established in sports like professional soccer. During high-speed sprinting, these strains predominantly occur at the end of the late swing phase. At this critical point, the hamstrings must function eccentrically to decelerate knee extension before rapidly transitioning to concentric function for propulsion. This rapid changeover, combined with a massive eccentric load, places the muscle fibers in a highly susceptible state for structural tearing (50, 51). Severe presentations of these injuries, such as proximal hamstring avulsions, have been clinically documented in elite Ultimate players. The common mechanism of injury for hamstring avulsion involves an extreme eccentric contraction of the hamstring muscles following forced hyperflexion of the hip with the knee fully extended. Within the specific context of Ultimate Frisbee, this catastrophic tendon failure can occur as a contact injury during aerial contests or layouts, such as when a player dives to catch the disc and an opposing player lands directly on top of them, forcing the hip and knee into this highly vulnerable biomechanical position (52). These strains are especially frequent in the later stages of competition when fatigue is more evident. Yen et al. (19) noted that frequent directional changes and jumps increase the risk of lower-limb injuries, with up to 30% of male injuries attributed to calf cramps. This suggests that high-intensity intermittent loading may induce muscle fatigue and contribute to the occurrence of injuries. Similarly, Hess et al. (15) reported a high prevalence of thigh strains among professional players, with injury incidence in the second half of games being 2.4 times higher than in the first half, indicating that fatigue-induced neuromuscular control deficits are critical contributors to both strains and sprains.

### 4.2 Bone injuries

Although bone injuries occur at a relatively low incidence in Ultimate Frisbee, they are most often associated with falls, unbalanced landings, or layouts involving high-impact collisions with the ground or other players (8, 15, 19). Swedler et al. (17) reported that fractures in Ultimate Frisbee typically occur when players sustain direct impacts while catching the disc or attempting to avoid a fall. Slaughter et al. (9) and Reynolds et al. (24) further noted that fractures of the hand and lower limbs are common during aggressive layouts or unstable landings. The biomechanics of these skeletal injuries can be closely mapped to collision and fall mechanisms in other impact sports. When players fall unsteadily or attempt to brace themselves during a failed layout, the instinctive protective mechanism often results in a Fall On Outstretched Hand. Although bone and structural joint injuries occur at a relatively low incidence in Ultimate Frisbee, they are most often associated with high impact collisions and aggressive maneuvers. The hands and digits are highly vulnerable during contested catches or when receiving high velocity throws. The direct impact of the rigid disc or accidental entanglement with another player frequently results in severe phalangeal fractures, interphalangeal joint dislocations, and tendon avulsions. These specific trauma patterns closely mirror the digital injuries sustained from direct ball impacts or forceful jersey grasping in collision sports including rugby and football (53). Furthermore, during a layout, players often land directly on the lateral aspect of the shoulder. This impact delivers a direct traumatic force to the superior aspect of the acromion while the arm is adducted, thereby driving the scapula inferiorly relative to the clavicle. This specific overloading mechanism is the primary etiology for the severe acromioclavicular joint separations commonly observed in young athletic populations (54). Although less frequent than soft-tissue injuries, bone injuries generally require longer recovery periods and, in severe cases, may compromise athletes' long-term performance and competitive capacity.

### 4.3 Neurological injuries

Although Ultimate Frisbee is classified as a non-contact sport, the risk of head impact remains non-negligible in high-intensity contests and aerial challenges. Lazar et al. (30) reported a lifetime prevalence of concussion as high as 26% among Ultimate athletes. In a prospective epidemiological study of collegiate Ultimate players, Swedler et al. (17) reported 29 concussions among female athletes, accounting for approximately 35% of all head and face injuries in this cohort. The primary mechanisms included head-to-ground contact during layouts and high-velocity disc impacts to the face or crown of the head, representing typical “non-contact” impact scenarios. Previous studies have shown that concussions in Ultimate Frisbee often result from head-to-ground contact during layouts, as well as head-to-body collisions during competitive plays (17, 19). Biomechanical evaluations of head impacts from collision sports elucidate this injury mechanism. When the head sustains a sudden impact against the ground or another player, the brain is subjected to severe linear and, more dangerously, rotational acceleration within the skull. This rotational acceleration produces profound shear forces that stretch and tear delicate neural fibers, ultimately leading to concussive episodes (55, 56). Alarmingly, previous research found that nearly half of concussed Ultimate athletes returned to play on the exact same day. The gravity of this issue is underscored by evidence from collision sports, which demonstrates that repeated concussive impacts and inadequate recovery periods are strongly associated with severe long-term neurodegenerative outcomes, including persistent post-concussion symptoms, early-onset cognitive impairment, and depression (57, 58).

### 4.4 Superficial tissue injuries

Superficial injuries, such as skin abrasions and soft-tissue contusions, are highly prevalent in Ultimate Frisbee, particularly affecting the upper limbs and trunk. Evidence indicates that layouts often involve extensive skin contact with the ground, resulting in abrasions or contusions of the knees, elbows, and shoulders. Marfleet et al. (23) identified abrasions as the most common type of skin injury in Ultimate Frisbee, typically caused by concentrated frictional forces during layouts. Such injuries are particularly common when technical execution is suboptimal and the body fails to dissipate impact forces through rolling (23). The severity and frequency of these superficial injuries are significantly influenced by the playing surface. For instance, variations in field conditions, such as the surface hardness and turfgrass shear strength, directly dictate the friction and impact forces experienced by athletes during a layout (59). Moreover, biomechanical comparisons across diverse playing surfaces, such as natural grass and beach sand, demonstrate that pitches with excessive stiffness and inadequate shock absorption impede smooth sliding mechanics, thereby amplifying epidermal shear forces and significantly elevating the incidence of severe friction burns and abrasions (60). While most superficial injuries are mild, their cumulative effects can impair athletic performance and disrupt training continuity.

Currently, research on injury types in Ultimate Frisbee has predominantly focused on soft-tissue injuries, with comparatively less attention given to high-risk conditions such as fractures and concussions, along with their clinical consequences and management pathways. This imbalance limits the integration of data across studies and hinders the development of comprehensive prevention strategies. Future studies should place greater emphasis on the mechanisms, long-term outcomes, and rehabilitation trajectories of severe injuries, and design targeted preventive and interventional measures tailored to the specific characteristics of Ultimate Frisbee and the progression of its injury patterns.

## 5 Injury prevention

### 5.1 Protective equipment

Johnson et al. (61) highlighted that footwear often serves as the primary “load-bearing point” under external forces, and its design and performance significantly impact structural stability. Since there are currently no sport-specific shoes designed for Ultimate Frisbee, players typically use football or American football cleats. These shoes are optimized for linear acceleration and impact resistance, but their inherent stud configuration, forefoot rigidity, and midfoot support are not fully compatible with the frequent lateral cuts, abrupt stops, and jump-landings characteristic of Ultimate Frisbee. Research specifically investigating footwear performance in Ultimate Frisbee indicates that approximately 88% of shoe damage occurs at the medial and lateral toe-box, which directly reflects the extreme mechanical loading concentrated in this region (61). To fully understand the injury mechanisms associated with this structural mismatch, it is critical to evaluate the cleat-surface interaction. While adequate translational traction is necessary for rapid acceleration, excessive rotational traction, particularly on artificial turf, constitutes a primary biomechanical culprit for non-contact injuries. When an athlete attempts a sudden directional change, high rotational traction can effectively lock the foot into the playing surface. This immobilization transfers immense torsional and shear forces directly to the ankle and knee joints, thereby severely increasing the risk of ACL ruptures and lateral ankle sprains (62, 63). Furthermore, the specific geometry of the cleats, such as the stud shape, profoundly influences plantar pressure distribution and the center of pressure excursion during lateral cutting maneuvers (64). During critical injury-prone moments like jump-landings, the approach angle of the ankle, specifically when positioned in combined plantarflexion and inversion, significantly alters the contact area between the boot and the surface. This reduction in contact area subsequently decreases safe translational traction and escalates the susceptibility to acute lateral ankle sprains (65, 66). Given that lower-limb injuries accounting for more than 60% of all cases (15, 17, 25), especially ankle and knee sprains, the structural mismatch of current footwear may increase the likelihood of non-contact injuries by limiting agility, reducing shock absorption during landings, and providing insufficient ankle support. Drawing from design advances in basketball, volleyball, and soccer, Ultimate-specific shoes should incorporate several key features. First, the stud shape and configuration must be optimized for multidirectional cutting and rapid starts, specifically by avoiding studs that are too long to prevent excessive ground penetration and hazardous rotational traction, or too short to provide sufficient translational traction (63). Second, footwear should include enhanced ankle-locking systems and lateral stabilizers to reduce the risk of inversion injuries (67). Finally, alternative outsole designs suitable for both natural grass and artificial turf are necessary. Future footwear development should integrate kinematic data and plantar pressure distribution specific to Ultimate actions, enabling biomimetic structural design and wear-testing to improve sport-specific adaptation.

### 5.2 Sport-specific physical conditioning

Integrating morphological and physical conditioning with optimal nutrition and systematic protocols, such as plyometrics and flexibility training, significantly enhances structural development and biomechanical efficiency. To effectively prevent injuries, however, conditioning programs must be rigorously aligned with the specific physiological and biomechanical demands of Ultimate Frisbee. Epidemiological surveys have shown that most injuries in Ultimate Frisbee are related to abrupt stops, cutting, and high-speed sprinting (10, 15). Thus, physical conditioning programs should be aligned with sport-specific demands, targeting strength, speed, endurance, and agility to support actions such as sprinting, directional changes, and precise disc-catching. Cheng et al. (27) found that 50.7% of surveyed athletes in Selangor regarded strength training as critical for injury prevention, as it improves explosive performance in sprinting and jumping while reducing lower-limb injury risk. Conditioning strategies should therefore match Ultimate's performance requirements.

Ultimate Frisbee matches impose high intermittent loads, with players sustaining average heart rates at 82% of maximum (160 ± 6 bpm), and spending 41.6% of total match time (approximately 22.5 minutes) above 90% of maximum heart rate (HRmax) (11). Crucially, players averaged 17.4 ± 5.7 sprints per game, with durations of 2.1 ± 0.5 s and recovery intervals of 226 ± 113 s (11). It is critical to note that these 226-s intervals do not represent passive recovery; rather, they are filled with continuous sub-maximal intense activities, including high-intensity running (14–20 km/h), rapid accelerations, and abrupt multidirectional decelerations. This is further compounded during tournament play, where athletes experience profound accumulated fatigue—evidenced by significant drops in relative exercise intensity and respiratory rate during matches on consecutive days (12, 13). Female athletes experience greater physiological stress, with significantly higher heart rates and blood lactate levels compared to men (7).These findings support integrating high-intensity interval training (HIIT) into Ultimate programs to enhance sprint performance, sustain workload, and reduce fatigue-related injuries (68). Specifically, Fartlek training—a continuous interval training method—has proven effective in enhancing the anaerobic sprint performance of Ultimate Frisbee players (69). Furthermore, utilizing Small-Sided Games (SSGs) with modified pitch sizes serves as a highly specific conditioning tool to safely replicate match demands and build aerobic capacity (70).

SAQ (Speed, Agility, Quickness) and stretch–shortening cycle (SSC) training are recommended to enhance lower-limb explosiveness and proprioceptive stability, supporting acceleration, jump performance, and cutting mechanics. Specifically, biomechanical evaluations of Ultimate Frisbee players reveal that isolated parameters including hamstring flexibility and soleus strength do not independently determine superior vertical jump performance and sprint velocity. This finding establishes that lower extremity conditioning must emphasize holistic stretch shortening cycle efficiency and integrated neuromuscular coordination rather than focusing exclusively on isolated muscular metrics (71). While widely used in soccer and basketball, targeted neuromuscular interventions are now being validated directly in Ultimate Frisbee (72, 73). For example, an 8-week modified FIFA 11 + training program significantly improved postural balance and proprioception in national-level Ultimate Frisbee players with functional ankle instability (74). Similarly, structured functional training effectively enhances postural balance in collegiate athletes, mitigating non-contact injury risk under fatigue (75). Additionally, specialized evaluations of Ultimate Frisbee athletes highlight that dedicated coordination and postural stability exercises are essential for maintaining center-of-gravity control during the sport's characteristic dynamic movements, thereby further reducing susceptibility to acute lower-limb trauma (76, 77).

Finally, physical conditioning plans must systematically account for specific positional demands. Handlers require prioritized aerobic endurance and agility, whereas cutters must focus on sprinting, acceleration and deceleration mechanics, and explosive strength. Furthermore, tailored training loads and recovery strategies remain especially crucial for female athletes to effectively mitigate physiological overload.

Crucially, physical conditioning should also integrate cognitive and ethical components unique to the sport. Evidence suggests that implementing attentional training programs can significantly enhance the decision-making quality of players during passing sequences, thereby reducing erratic movements that lead to collisions (78). Finally, physical conditioning plans may systematically account for specific positional demands. Handlers require prioritized aerobic endurance and agility, whereas cutters must focus on sprinting, acceleration and deceleration mechanics, and explosive strength. Furthermore, tailored training loads and recovery strategies remain especially crucial for female athletes to effectively mitigate physiological overload.

### 5.3 Tactical training

To effectively mitigate injury risks during high-intensity play, athletes must achieve technical proficiency alongside advanced tactical awareness. Athletes should master multiple throwing techniques, including backhand, forehand, and hammer throws, to ensure precise control over disc trajectory and force, thereby reducing the likelihood of erratic plays that lead to dangerous collisions (79, 80). Beyond individual mechanics, tactical training must address passing coordination, spatial organization, and collective decision-making (4, 16). Offensive systems such as the vertical stack, horizontal stack, and zone offense are widely applied to structure team movement and optimize field spacing (81, 82). The vertical stack offense, for instance, relies on coordinated cutting lanes to maintain a clear “open side” for handlers. Mastery of timing and spacing within these systems is essential to avoid congested field areas, particularly in the end zone, where high-speed collisions and contested aerial catches are most frequent (80, 83). Defensively, the implementation of zone and hybrid schemes requires rapid collective rotations to occupy open spaces effectively. These structured defensive movements can proactively reduce the probability of accidental high-impact impacts by preventing multiple defenders from converging on a single offensive player (16, 82). Furthermore, observational analyses of elite competitions, such as the Colombian National Championship, emphasize that systematically evaluating these technical and tactical characteristics during high-stakes matches is crucial for understanding how teams maintain structural integrity under competitive pressure (84). By continuously analyzing real-match tactical execution, teams can refine their spatial organization to minimize chaotic, injury-prone transitions.

A critical component of tactical injury prevention is the cognitive demand placed on players during rapid transitions and set plays. Research indicates that an athlete's decision-making, particularly pass selection under defensive pressure, is heavily influenced by their attentional focus. Implementing structured attentional training programs has been shown to significantly enhance the decision-making quality of Ultimate Frisbee players. By improving situational awareness and environmental cue processing, such training allows players to select safer passing lanes and anticipate hazardous movement patterns before they result in contact (78). Furthermore, adhering to the “Spirit of the Game” (SOTG)—the sport's foundational principle of fair play and self-officiating—acts as an intrinsic tactical deterrent against dangerous play and intentional fouling, which is vital for maintaining player safety in a non-contact environment (16).

The successful execution of complex tactical maneuvers depends heavily on superior neuromuscular control and postural stability. Evidence demonstrates that structured functional training and modified neuromuscular protocols, such as the FIFA 11+, significantly improve postural balance and proprioception in Ultimate Frisbee athletes (74, 75). Enhanced balance allows players to maintain better motor control during the abrupt decelerations, sharp pivots, and jump-landing sequences required by advanced offensive and defensive tactics, thereby mitigating the risk of non-contact ankle and knee injuries (77). By integrating these cognitive and physiological interventions, tactical training becomes a primary tool for both performance optimization and systematic injury risk reduction.

### 5.4 Application of smart technologies

The integration of advanced data analytics and wearable technologies offers a transformative framework for performance optimization and injury mitigation in Ultimate Frisbee. State transition modeling (STM) has emerged as a particularly promising quantitative method for analyzing tactical efficiency and disc flow patterns. Lam et al. analyzed data from 14 AUDL games using STM and identified significant variances in flow efficiency across field zones, concluding that midfield organization and front-field decision-making are primary determinants of scoring success (85). These models provide coaches with actionable insights to optimize offensive structures and identify high-probability scoring pathways, thereby reducing disorganized play that contributes to collision risks. Beyond tactical modeling, machine learning algorithms evaluate historical game statistics to estimate team win probabilities in the Ultimate Frisbee Association (UFA), providing a quantitative basis to personalize training priorities and workload distribution based on empirically derived success predictors (86). Comprehensive statistical analyses of longitudinal game data from elite women's programs further classify player performance metrics and opponent characteristics. This high-resolution data identifies the exact team features driving competitive success, establishing a data-driven foundation for injury-conscious roster management and tactical refinement (87).

Wearable sensors combined with sophisticated algorithms are increasingly utilized for real-time activity recognition and biomechanical assessment. Link et al. demonstrated the efficacy of using wrist-worn inertial measurement units (IMUs) in conjunction with convolutional neural networks (CNNs) and transfer learning to automatically classify fundamental throwing techniques with an accuracy of 89.9% (9). Complementing sensor-based monitoring, non-invasive computer vision frameworks utilize deep learning for automated biomechanical assessment. Human activity recognition models, including the UltiVision system, classify specific kinematic errors in forehand throws—specifically the absence of wrist flicks or foot pivots—to establish an automated coaching foundation that delivers immediate technical correction and prevents the development of hazardous throwing mechanics (88). Beyond upper-extremity mechanics, wearable movement sensors directly quantify the biomechanical characteristics of in-game lower-limb maneuvers. Slaughter and Adamczyk (9) reconstructed lower-body kinematics using inertial sensors worn by female Ultimate Frisbee athletes during competitive matches, identifying 422 distinct cutting maneuvers. Their analysis revealed a mean ground-contact knee flexion of 34° alongside approach speeds of 3.4 m/s. Tracking these specific quantitative characteristics highlights high-risk movement patterns, particularly fast cutting velocities combined with reduced knee flexion angles, that severely elevate ACL injury risk in female athletes.

The diagnostic application of artificial intelligence is further revolutionizing injury risk prediction through the adaptation of established athletic models. Research in soccer demonstrates that machine learning algorithms, including decision trees, support vector machines (SVMs), and XGBoost, predict overuse injuries with an accuracy of approximately 88% when integrated with GPS data (89). Furthermore, Rossi et al. (90) demonstrated the feasibility of real-time training load monitoring and injury risk estimation, a methodology directly adaptable to Ultimate Frisbee. By utilizing IMUs and GPS to capture running distance, sprint frequency, and acceleration patterns, these models effectively quantify fatigue and personalize training loads to prevent physiological overload (89). Beyond muscular fatigue, diagnostic hardware extends to concussion management. While instrumented smart mouthguard systems are currently utilized in contact sports including rugby to monitor head impact events via high-frequency accelerometers and digital filters, this technology holds significant potential for future expansion into Ultimate Frisbee (91, 92).

Finally, longitudinal monitoring of sport-specific adaptations provides early warnings for overuse conditions. Elite Ultimate Frisbee players often exhibit specific shoulder strength imbalances and mobility adaptations on the dominant throwing side, patterns consistent with those observed in other overhead sports (93–95). Future applications of AI and kinematic sensors facilitate angle-specific ratio assessments during the acceleration and follow-through phases of a throw, revealing load transfer patterns that precede shoulder injuries. Ultimately, the continuous evolution of smart technologies—ranging from machine learning algorithms evaluating tactical loads and computer vision systems correcting throwing mechanics, to wearable sensors monitoring real-time biomechanics and head impacts—creates a comprehensive diagnostic ecosystem. This integration transitions Ultimate Frisbee training and injury prevention from reactive treatments to proactive, data-driven management, ensuring sustained athletic performance and optimal joint health.

### 5.5 Clinical implications for rehabilitation professionals

Rehabilitation professionals play a critical role in managing Ultimate Frisbee injuries and orchestrating safe return to play transitions. Given the unique biomechanical demands of the sport, conventional rehabilitation protocols should be adapted to address specific athletic requirements, particularly extreme eccentric deceleration, multidirectional cutting, and frequent jump landings. For lower extremity joint rehabilitation, clinical evidence indicates that generalized physical therapy is often insufficient. Specifically, regarding knee injuries, a recent randomized controlled trial focusing exclusively on Ultimate Frisbee athletes demonstrated that individualized exercise prescriptions yield superior clinical outcomes. These tailored interventions should incorporate progressive phases of joint mobility restoration, varied resistance muscle strengthening ranging from isometric to isokinetic loads, and dynamic balance training that is continuously adjusted based on the athlete's specific functional deficits and pain thresholds. Implementing such highly specific protocols significantly accelerates knee joint functional recovery and alleviates pain. Furthermore, these personalized approaches effectively reduce systemic inflammatory responses and enhance psychological resilience during the rehabilitation process (5).

Regarding the high incidence of hamstring strains sustained during high-speed sprinting and abrupt deceleration in Ultimate Frisbee, rehabilitation professionals can prioritize eccentric strength restoration and functional sprinting mechanics. Clinical evidence supports the early introduction of high-intensity eccentric loading in the rehabilitation process. Implementing exercises including Nordic hamstring curls safely accelerates the lengthening of muscle fascicles and restores the tissue's capacity to absorb massive eccentric forces, which effectively mitigates the established risk of strain recurrence (96–98). Because the biomechanical demands of offensive cutting and defensive tracking in Ultimate Frisbee closely mirror the kinematics of track-and-field sprinting, rehabilitation protocols should carefully bridge the gap between isolated strengthening and sport-specific locomotion. Kinematic analyses suggest that rehabilitative exercises should closely simulate the precise muscle activation patterns observed during the late swing phase of sprinting. By integrating hip-extension dominant movements alongside progressive running programs, physical therapists can ensure that the biceps femoris is adequately prepared for the maximum-velocity deceleration required on the ultimate pitch (99, 100). In instances of catastrophic soft-tissue failure, specifically proximal hamstring ruptures resulting from sudden slips or awkward collision landings that force the hip into extreme flexion while the knee remains extended, physical therapists are advised to implement a meticulously phased surgical rehabilitation pathway. This clinical progression initially prioritizes tissue healing by strictly restricting hip flexion and knee extension ranges to protect the surgical repair. Subsequent phases introduce submaximal isometric loading and progressive concentric strengthening, which eventually transition into delayed high-load eccentric exercises once structural integrity is confirmed. The final rehabilitation stage culminates in sport-specific multidirectional agility drills to restore dynamic neuromuscular control (101, 102). Ultimately, the criteria for return to play should shift from arbitrary time-based paradigms to objective functional assessments. Global surveys of elite professional soccer programs and contemporary sports medicine guidelines dictate that athletes are recommended to receive medical clearance for competition only once they fulfill specific functional criteria. These rigorous standards, which are directly adaptable to Ultimate Frisbee, require athletes to demonstrate symmetric eccentric hamstring strength, execute pain-free maximal sprinting, and possess the psychological readiness to perform unpredictable reactive maneuvers without apprehension (103).

Because Ultimate Frisbee currently lacks sport-specific randomized controlled trials for lateral ankle sprain rehabilitation, physical therapists can extrapolate evidence-based protocols from sports with analogous biomechanical demands. In disciplines requiring rapid directional shifts and frequent jump-landings, clinical evidence demonstrates that structured neuromuscular and proprioceptive training programs significantly enhance joint position sense and dynamic balance while effectively reducing injury recurrence rates (104–106). Furthermore, studies involving soccer athletes reveal that integrating cognitive interventions including motor imagery alongside conventional physical therapy accelerates the recovery of postural control by mitigating central nervous system deficits (107). Prophylactic strategies emphasizing consistent proprioceptive exercises combined with external supports are highly effective for preventing recurrent injuries in basketball and other high-demand activities (108, 109). Consequently, systematically applying these extrapolated protocols to Ultimate Frisbee is highly recommended to effectively restore dynamic joint stability. Similarly, to ensure safe return to play, practitioners are encouraged to adopt objective clinical metrics established in professional soccer and related disciplines rather than relying on subjective symptom resolution. Utilizing functional assessments including the Star Excursion Balance Test or the Y-Balance Test accurately identifies residual reach asymmetries and dynamic postural control deficits (110, 111). Satisfactory performance on these adapted metrics serves as a crucial clinical indicator that the athlete's sensorimotor system is adequately prepared for the unpredictable landing environments characteristic of Ultimate Frisbee.

Regarding sport related concussions resulting from aerial collisions, clinicians are advised to implement a stepwise return to play protocol. Medical consensus suggests a gradual progression of physical activity, ensuring the athlete remains entirely asymptomatic at each stage before advancing to more complex tasks (112). Crucially, clinical assessments should account for the intense physical stress of the sport prior to final medical clearance. Research indicates that evaluating neurocognitive function following high intensity intermittent exercise provides a highly accurate assessment of an athlete's readiness. Integrating post exercise cognitive testing helps identify any residual deficits that may only emerge under physical fatigue, ensuring that athletes can safely tolerate the cardiovascular and cognitive demands of Ultimate Frisbee before fully returning to competition (113).

## 6 Conclusions and future perspectives

### 6.1 Conclusions

As a rapidly evolving and high-intensity team sport, Ultimate Frisbee is characterized by an injury profile that is anatomically specific, diverse in pathology, and biomechanically complex. Current epidemiological evidence consistently demonstrates a predominance of lower-limb injuries, specifically lateral ankle sprains, anterior cruciate ligament tears, and severe hamstring strains. These pathologies are primarily driven by the extreme eccentric and torsional forces generated during non-contact sprinting, rapid deceleration, and multidirectional cutting. Concurrently, severe upper-body and neurological traumas, including fractures resulting from falling on an outstretched hand, acromioclavicular separations, and concussions, represent high-risk consequences of layouts, aerial contests, and incidental collisions.

Effectively mitigating these risks requires transcending generic athletic conditioning. The synthesis of current evidence advocates for a multidimensional and sport-specific prevention paradigm. This framework includes correcting the cleat-surface mechanical mismatch through optimized footwear, implementing targeted neuromuscular protocols including speed-agility-quickness drills and the modified FIFA 11+ program, and leveraging tactical spatial organization techniques like State Transition Modeling alongside the intrinsic Spirit of the Game principles to minimize hazardous collisions. Furthermore, the integration of smart technologies, ranging from sensor-driven biomechanical assessments and computer vision for technical correction to machine learning for workload prediction, ushers in a proactive and data-driven era of athlete health management. Finally, post-injury management must transition toward individualized, sport-specific rehabilitation protocols and utilize objective functional metrics to dictate safe return-to-play, thereby effectively mitigating the risk of reinjury.

### 6.2 Research gaps and future directions

Despite these promising advancements, substantial methodological and demographic gaps remain in the current literature. The existing epidemiological landscape is heavily reliant on retrospective self-reporting, resulting in inconsistent tracking of true athlete-exposures. Moreover, current research disproportionately focuses on male or collegiate cohorts, leaving a critical void in understanding sex-specific vulnerabilities, particularly the elevated anterior cruciate ligament injury risk among elite female athletes, and the biomechanical consequences of accumulated fatigue across multi-day tournaments. Furthermore, direct causal links between specific tactical behaviors and injury mechanisms have not been systematically established through long-term data.

To bridge these gaps and establish a robust safety framework for Ultimate Frisbee, future research must prioritize the following directions: (1) Epidemiological Standardization: Establish multicenter and prospective injury registries utilizing standardized definitions to accurately quantify true incidence rates across different sexes, age groups, and competitive tiers. (2) Sport-Specific Equipment Innovation: Transition from the adaptation of traditional soccer or American football cleats to the biomechanical development and rigorous clinical validation of Ultimate-specific footwear, optimizing multidirectional traction and shock absorption to prevent non-contact joint trauma. (3) Interventional Validation via Controlled Trials: Conduct large-scale randomized controlled trials based on sports biomechanics and load monitoring to empirically validate the efficacy of multimodal prevention programs, including the integration of high-intensity interval training with proprioceptive exercises, on actual injury reduction. (4) Advanced Technological Integration: Expand the application of artificial intelligence, big data analytics, and wearable sensors in real-match scenarios to provide coaching and medical staff with predictive models for personalized load management, early risk identification, and tactical optimization.

By integrating these multidisciplinary efforts, the sports medicine community can transition Ultimate Frisbee injury management from reactive treatments to proactive prevention, ultimately safeguarding athlete longevity and elevating the global standard of competition.

## 7 Limitations of the review

While this comprehensive review synthesizes three decades of epidemiological and biomechanical research on Ultimate Frisbee, several methodological limitations must be acknowledged. First, the significant heterogeneity in injury definitions across the included primary studies precludes the execution of a quantitative meta-analysis. Because researchers utilized varying criteria ranging from strict time-loss metrics to broader medical-attention definitions, the synthesized incidence rates can only be presented descriptively rather than statistically pooled. Second, the reliance on retrospective cross-sectional surveys in a substantial portion of the available literature introduces inherent recall bias. This methodological constraint likely leads to the underreporting of minor, non-time-loss injuries while overemphasizing memorable catastrophic events. Finally, because direct interventional evidence specifically targeting Ultimate Frisbee remains scarce, many of the proposed prevention and rehabilitation strategies are predominantly extrapolated from analogous high demand field sports. Consequently, the sport specific efficacy of these extrapolated protocols warrants future empirical validation through dedicated randomized controlled trials.
